# State Medical Service

**Published:** 1918-12-07

**Authors:** 


### STATE MEDICAL SERVICE.

THE November *Practitioner* has an article by Dr. William A. Brend, on State Medical Service. Many suggestions that have been made are reviewed. The suggestions display diversity of opinion, but, as Temporary Major Brend, M.D. Lond., points out, there are agreements. It is agreed on all sides that the possibilities of diagnosis and treatment that the rapid increase of knowledge has given us in some way must be placed at the service of all the public. It is agreed that means to give nursing to all must be found. It is agreed that hospitals should receive State subsidies but remain voluntary. If the agreement is not absolute, it is a majority opinion. The object of many of the suggestions is to reconcile the continuance of private practice with a National Medical Service.

If there be a State Medical Service there must be much interference with the way in which every doctor does his work, whether he be a State medical officer on full-time or on part-time, or not a State medical officer at all. If there is to be interference there does not seem to be any advantage in that interference originating locally rather than centrally. There are two courses, either individualistic practice or a State Medical Service. Each has its drawbacks and advantages. It is for the nation to decide between the two systems. If the nation wants a State Service it must accept the drawbacks with the gains.

It is hard to distinguish in his remarkably judicial article which are Dr. Brend's own views on the subject and which are the opinions of others quoted for review. It seems that Dr. Brend thinks a good solution would be local Medical Services in some places, differing according to the locality, and no State Medical Service but individual practice in others. It is very hard to see how such distinctions could be made, and the system would be a patchwork. One obvious drawback is that statistics of value could not be compiled. Dr. Brend clearly sees the difficulty of organising a State Medical Service only for those whose income is below a certain level, and the difficulties that would, and do now, arise from a man having two sets of clients, State Service patients paid for at so much a head and private patients. Dr. Brend has evidently thought so much about a State Medical Service, has studied the question so earnestly, and has seen both sides so clearly, that a pronouncement from him on the form a State Service might usefully take would be a valuable contribution. The point to keep in view is that a State Medical Service must be demanded by the people of Great Britain before it can come into being. It is not a demand of the medical profession, as yet. In fact, at present, on the whole, the medical profession is against the suggestion. But the medical profession must be ready to meet the demand, if it come. Ready with plans, goodwill and determination to meet the wishes of the community.

